# Increased Susceptibility for Thromboembolic Events versus High Bleeding Risk Associated with COVID-19

**DOI:** 10.3390/microorganisms10091738

**Published:** 2022-08-29

**Authors:** Cristina Tudoran, Dana Emilia Velimirovici, Delia Mira Berceanu-Vaduva, Maria Rada, Florica Voiţă-Mekeres, Mariana Tudoran

**Affiliations:** 1 Department VII, Internal Medicine II, Discipline of Cardiology, University of Medicine and Pharmacy “Victor Babes” Timisoara, E. Murgu Square, Nr. 2, 300041 Timisoara, Romania; 2 Center of Molecular Research in Nephrology and Vascular Disease, Faculty of Medicine, University of Medicine and Pharmacy “Victor Babes” Timisoara, E. Murgu Square, Nr. 2, 300041 Timisoara, Romania; 3 County Emergency Hospital “Pius Brinzeu”, L. Rebreanu, Nr. 156, 300723 Timisoara, Romania; 4 Academy of Romanian Scientists, Ilfov Str. Nr. 3, 50085 Bucuresti, Romania; 5 Department VI, Internal Medicine and Ambulatory Care, Prevention and Cardiovascular Recovery, University of Medicine and Pharmacy “Victor Babes” Timisoara, E. Murgu Square, Nr. 2, 300041 Timisoara, Romania; 6 Department XIV, Microbiology, University of Medicine and Pharmacy “Victor Babes” Timisoara, E. Murgu Square, Nr. 2, 300041 Timisoara, Romania; 7 Department of Morphological Disciplines, Faculty of Medicine and Pharmacy, University of Oradea, 1 Universitatii Street, 410087 Oradea, Romania

**Keywords:** COVID-19, thromboembolic events, arterial thrombotic events, venous thrombotic events, major bleedings, hematomas, alterations of hemostasis, anticoagulant therapy

## Abstract

The infection with the SARS-CoV-2 virus is associated with numerous systemic involvements. Besides the severe respiratory injuries and cardiovascular complications, it became obvious early on that this disease carries an increased risk of thromboembolic events, but a higher propensity for bleedings as well. We researched the medical literature over significant PubMed published articles debating on the prevalence, category of patients, the moment of occurrence, and evolution of venous thromboembolism (VTE), but also of venous and arterial “in situ” thrombosis (AT), and hemorrhagic events as well. Most researchers agree on an increased prevalence of thromboembolic events, ranging between 25 and 31% for VTE, depending on the analyzed population. For AT and hemorrhagic complications lower rates were reported, namely, about 2–3%, respectively, between 4.8 and 8%, occurring mostly in older patients, suffering from moderate/severe forms of COVID-19, with associated comorbidities. It is important to mention that patients suffering from hemorrhages frequently received thromboprophylaxis with anticoagulant drugs. As a consequence of thromboembolic and hemorrhagic complications which are both important negative prognostic factors, the evolution of patients infected with the SARS-CoV-2 virus is aggravated, determining an augmented morbidity and mortality of this population.

## 1. Introduction

The infection caused by the severe acute respiratory syndrome CoV-2 (SARS-CoV-2) virus has persisted for almost three years, inducing the most significant global pandemic of the last century named COVID-19, which has shaken the lives of millions of people and health systems worldwide [1,2]. Although the virus primarily affects the lungs, it became obvious from the early stages that this illness is associated with an increased risk for thromboembolic events such as deep vein thrombosis (DVT), pulmonary embolism (PE), and even arterial in situ thrombosis (AT) in some cases [3,4,5]. Numerous scientific studies [6,7,8], and even significant meta-analyses [6,9,10,11,12], have been published on this topic, analyzing various populations, most of them referring to the acute phase of the disease, often in anticoagulated patients [13,14]. Conversely, the alteration of hemostasis and the increased risk of bleeding observed in COVID-19 patients are less debated in the medical literature [15,16]. An increased prevalence of disseminated intravascular coagulopathy (DIC) and/or hemorrhagic complications has been described in critically ill patients [9,17,18,19], and also several cases with subcutaneous hematomas (SH), occurring during this illness and considered to be associated with the concomitant anticoagulant therapy (ACT) has been reported [17,20,21].

**Figure 1:**
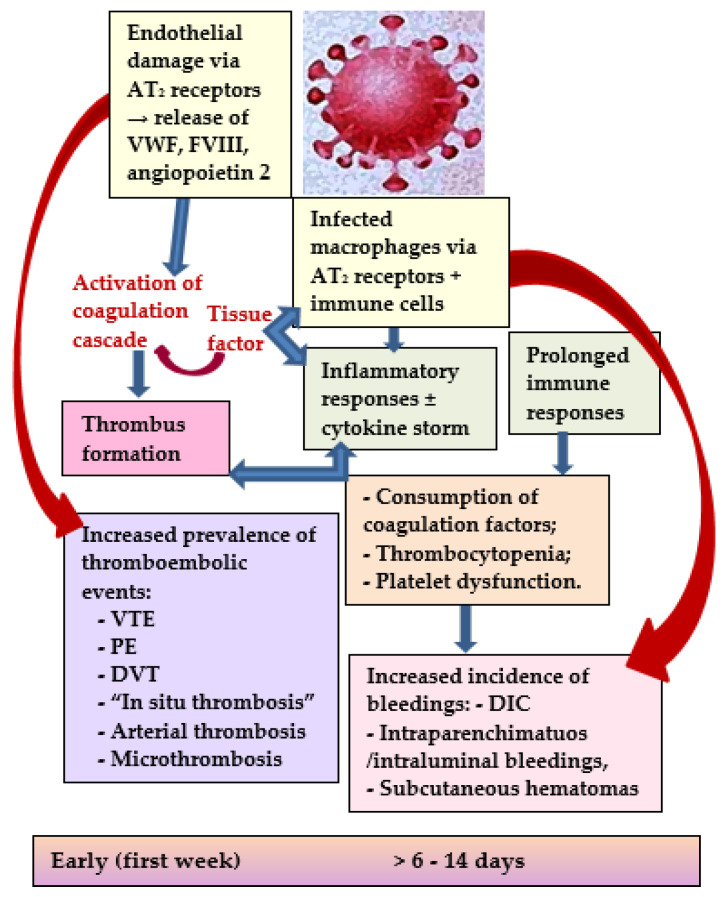
Schematic presentation of pathophysiological pathways responsible for the occurrence of thromboembolic and bleeding complications in COVID-19. Legend: AT2-angiotensin-II; VWF-von Willebrand factor; FVIII-factor VIII; VTE-venous thromboembolism; PE-pulmonary embolism; DVT-deep vein thrombosis; DIC-disseminated intravascular coagulopathy.

The involvement of the cardiovascular system, particularly the increased risk of thromboembolic events, is considered to contribute significantly to the increased morbidity and mortality of COVID-19 patients. There are numerous pathophysiological mechanisms considered to be responsible for the thrombosis and coagulation disturbances observed in this disease as presented schematically in Figure 1: (a) an endothelial dysfunction, induced by the coupling of the virus with the specific angiotensin-II receptors of the endothelial cells resulting in an increased release of the vasoconstrictor angiotensin-II, and reduced levels of the angiotensin I which acts as a vasodilator [2,22,23,24,25,26]; (b) an exaggerated inflammatory response observed especially in most patients with severe COVID-19, expressed by abnormally elevated levels of proinflammatory cytokines such as interleukin 6 (IL-6), IL-17A, and tumor necrosis factor, generating the so called “cytokine storm” which mediates the activation of platelets and the coagulation cascade [22,23,24,25]; (c) another essential element of the coagulation cascade, the tissue factor, may be exposed by damaged endothelial cells or by monocytes, but it could also be activated by the elevated cytokines level, frequently resulting in thrombin production and consequent alveolar and capillary thrombosis; (d) moreover, prolonged inflammation, as a consequence of severe infection or sepsis, induces alterations of both coagulation and fibrinolysis by multiple pathways, such as reducing the antithrombin and protein C, and determining the elevation of plasminogen activator inhibitor-1 (PAI-1), and, thus, activating the coagulation cascade with the inhibition of fibrinolytic reaction resulting in increased tendency to thrombosis [4,15,23].

Additional factors include a multitude of well-known DVT risk factors, including platelet activation, severe hypoxemia, immobilization, mechanical ventilation, and the use of central venous catheters, all contributing to a prothrombotic state in COVID-19 [3,7,12]. In several scientific papers, there is a consensus that thromboembolic events occur more precociously in the course of COVID-19. The administration of anticoagulants is recommended to counterbalancethese hypercoagulability and prothrombotic tendencies, [3,9,27,28] and even guidelines have been issued on this indication [29,30]. On the other hand, multiple factors such as thrombocytopenia, hypofibrinolytic status, and depletion of coagulation factors, that render patients with COVID-19 prone to bleeding, initiate their action later, after one to three weeks, not to mention the administration of anticoagulants such as thromboprophylaxis [15,17,30,31] even in therapeutic doses. Moreover, considering the increased bleeding risk in critically ill COVID-19 patients favored by thrombocytopenia/platelet dysfunction and/or coagulation factor scarcity, or both [23,32], which are frequent occurrences in this population, it becomes more and more difficult to establish an appropriate, integrative, anticoagulant prophylaxis regimen [33]. It is thus understandable why there are discrepancies among various studies, especially concerning the doses and duration of the anticoagulant therapy for the prevention and therapy of thromboembolic and bleeding events in patients with COVID-19 [29,30,33].

## 2. Materials and Methods

**Figure 2:**
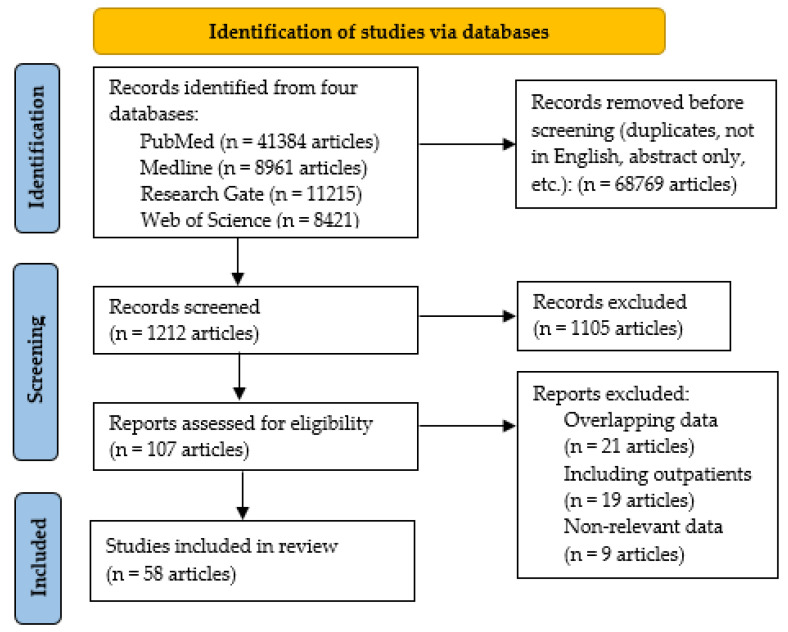
PRISMA flowchart of the medical articles selection.

To identify suitable articles for our review, we searched in the medical literature for scientific papers published in English between January 2020 and April 2022 on Pub-Med, Medline, Research Gate, and Web of Science, concerning thromboembolic and bleeding events that occurred in patients hospitalized for COVID-19. We employed keywords such as COVID-19, SARS-CoV-2 infection, thromboembolic events, venous thromboembolism (VTE), DVT, PE, AT, major bleedings, IDC, hematomas, alterations of hemostasis, and ACT. We employed the NIH (National Library of Medicine) and its Medical Subject Headings (MeSH) browser to select suitable MeSH terms for our keywords. Initially, we identified 69,981 articles in the four employed databases, and of them 68,769 were excluded before screening, Figure 2. During the screening process, the remaining 1212 articles were individually assessed by two authors (CT and MT), and other 1105 records were excluded (no full-text available in English, conceptual or epidemiologic research, not referring to hospitalized patients). The remaining 107 articles were reanalyzed, and after excluding another 49, the remaining 58 articles were included in this review.

The main objective of our study was to evaluate the prevalence of thromboembolic events, AT, and bleeding complications in patients hospitalized with COVID-19. A second purpose was to observe particularities related to the evolution and therapy of these populations. All full texts of the selected articles were reviewed for avoiding the risk of biases (ROB). For analyzing thromboembolic events, we selected meta-analyses and observational studies, such as cohort and cross-sectional studies, debating over more than 10 patients hospitalized for COVID-19. Considering the lower prevalence of AT and hemorrhagic complications, several representative articles presenting a series of case reports were also taken into consideration and presented in the results in a separate section. Articles debating over the pathophysiologic mechanisms of thrombosis and coagulopathies in COVID-19, and over histopathological and laboratory data, were employed for elaborating a comprehensive background in the discussions.

This study was authorized by the Scientific Research Ethics Committee of our hospital (No. 206/7.09.2020).

## 3. Results

### 3.1. Thrombotic Events in the Acute Phase of COVID-19

Overall, thromboembolic events were described more frequently in the medical literature, most researchers agreeing that VTEs have an earlier onset (in the first week). These events aggravate the evolution of patients hospitalized for COVID-19, despite of precocious thromboprophylaxis, and explain, at least partially, the increased mortality associated with this disease. In our review, we selected 30 relevant scientific papers of which were 8 meta-analyses, 15 cohort studies, and 1 prospective autopsy cohort study, as well as 6 retrospective observational case series debating on the prevalence of AT, the results being summarized in chronological order in Table 1 and Table 2. We analyzed only thromboembolic events described in hospitalized individuals and noticed that the reported prevalence varied largely between studies, depending on the analyzed population, the severity of the SARS-CoV-2 infection, and other factors, the risk being the highest in the intensive care units (ICU), among the critically ill patients [34].

A recent cohort study [35] evidenced a prevalence of 3.45% for VTEs, these events being more frequent in elderly men with associated comorbidities and increasing the mortality in COVID-19. VTEs were more frequent than AT, despite ACT (prophylaxis with LMWH).

A large systematic review and meta-analysis from 2021, analyzing 47 studies and 18,093 patients, indicated a 17.3% overall incidence of VTE among hospitalized COVID-19 patients, and ~2/3 of these events were reported as being DVT, while subgroup analysis revealed a much higher pooled prevalence of VTEs for patients admitted in ICUs in comparison to those from general wards (27.9% vs. 7.1%, respectively) [10]. Another meta-analysis of 12 studies, patients being under thromboprophylaxis, with low-molecular-weight (LMWH) or unfractionated heparin (UFH), still indicated a 31% pooled incidence of VTEs for ICU cases [36].

**Table 1:** Medical literature important studies referring to venous thrombotic events (VTE) in hospitalized patients.

Authors/Year	Study Type	No. of Studies/Patients	ICU	VTE	Prophylaxis AC	Comments
Lee et al./2022 [37]	Multicenter retrospective study	3531	ICU/ward	6.68%	88.55%	Increased hospitalization
Parks et al./2022 [38]	Multicenter cross-sectional study	1121	ICU/Ward	8.8%	86%	2.1% AT; 4.1% major and 4.1% minor bleedings
Tamayo-Velasco et al./2022 [35]	Retrospective cohort study	2894	10.85% ICU	3.45%	100%	1.8% AT
Boyd et al./2022 [39]	Retrospective study	128	All ICU	30%	98.18%	Increased risk of VTE despite prophylaxis
Ontiveros et al./2022 [40]	Retrospective case series	1/36	69% ICU	1.8% (36 cases out of 2000)	97.22%	Overall mortality was 55.6%.
Jimenez et al./2021 [10]	Meta-analysis	47 out of 49 studies/18,093	ICU/Ward	17.3%	100%	Bleedings prevalence 3.9%
Fontelo et al./2021 [11]	Meta-analysis	19/2554	ICU/Ward	28%	93.5%	Increased prevalence in ICU cases
Oba et al./2021 [41]	Retrospective single-center study	1/516	ICU/Ward	4.26	100%	3.8% AT
Munoz Rivas et al./2021 [28]	Retrospective single-center study	1/1127	Non-ICU	6.1	100%	1.6% AT
Perepu et al. [42]	Randomized control study	176	ICU/Ward	13%	100%	2% major bleedings
Hasan et al./2020 [36]	Meta-analysis	12/899	ICU/Ward	31%	100%	High prevalence of VTE despite prophylaxis
Piazza et al./2020 [43]	Retrospective cohort study	1/1114	15.26% ICU	27% ICU 2.2% non-ICU	89.4%	2.6% AT
Bilaloglu et al./2020 [44]	Retrospective cohort study	1/3334	24.86% (829 ICUpatients)	6.2%	LMWH prophylaxis	11.1% AT
Malas et al./2020 [6]	Meta-analysis	42/8271	ICU/Ward	21%	Almost all patients	2% AT; higher mortality
Fontana et al./2020 [34]	Meta-analysis	11/1369	6 studies only ICU	General: 4.4–8.2%. ICU: 35.3%	~100%	Higher prevalence in ICU, up to 53.8%
Chi et al./2020 [45]	Meta-analysis	11/1981	5 studies only ICU, 6 studies only ward (10%-38% needed ICU)	23.9% (11.9% DVT/11.9% PE)	100%	VTE-30.4%-ICU 13% in ward. PE: 15.7% in ICU, 2.4% in non-ICU
Wang et al./2020 [46]	Meta-analysis	28/4138	Reported for 20 studies	16% (all DVT)	Between 30 and 100%	23% ICU vs. 5% in non-ICU; China 30%/13% in western countries
Potere et al./2020 [9]	Meta-analysis	44/14.866	34% ICU	34% in ICU; 15% non-ICU	Not Reported	Overall mortality 10%
Klok et al./2020 [47]	Cohort study	184	100% ICU	31%	100%	AT 1.6%
Whyte and al./2020 [48]	Cohort study	1477	ICU/Ward	27%	100%	5.4% PE non-ICU 16.7–47% ICU
Lodigiani et al./2020 [49]	Retrospective cohort study	388	ICU/Ward	27.6% ICU 6.6% ward	100%	2.5% AT 2.1% bleeding
Trimaille et al./2020 [50]	Cohort study	289	Ward	17%	89.3%	Increased likelihood of ICU stay
Leonard-Lorant et al./2020 [51]	Retrospective cohort study	1/1696	45.28% overall (75% of cases with VTE)	30% (all PE)	46.2% overall	Thorax angio-CT study
Wichmann et al./2020 [52]	Prospective autopsy cohort study	1/12	41.66% (5 died after treatment in ICU)	58% (7 cases had DVT and PE in 4)	Not Reported	DVT and PE were found at autopsy in 4 patients

Legend: ICU-intensive care unit; VTE-venous thromboembolism; AC-anticoagulant; AT-arterial thrombosis; DVT-deep vein thrombosis; LMWH-low-molecular-weight heparin; MI-myocardial infarction; NA-not applicable; PE-pulmonary embolism.

The above-mentioned data are based more on clinical and laboratory investigations. By employing more specific procedures, the incidence was even higher. An analysis of 101 COVID-19 admissions who underwent duplex ultrasound (DUS) for clinical suspected DVT (onset of symptoms varied widely, although 73.8% of definite diagnoses were obtained in the first 2 weeks of admission), showed that 41.58% were positive for DVT, 6.93% had superficial thrombophlebitis and, moreover, 23.76% demonstrated PE (mostly involving peripheric, small caliber, pulmonary artery vessels), though only 7.92% had PE and concomitant, associated DVT, suggesting that 2/3 of PEs developed in absence of a diagnosed DVT, implying another mechanism of primary thrombosis instead of embolism [53]. Considering the 101 TEs, 70% were already diagnosed at the admission in the hospital, this determining those patients to attend the emergency department. In an autopsy cohort study, findings of COVID-19 fatalities suggested that venous thrombotic events may reach higher values of nearly 60% [52].

**Table 2:** Medical literature studies referring to arterial thrombotic events in hospitalized patients.

Authors/Year	Study Type	No. of Studies/Patients	ICU	AT	Prophylaxis	Commentary
Ilonzo et al./2021 [54]	Retrospective case series	1/21 hospitalized	Not reported	76.2%	76.2% of patients on antiplatelet and 19.1% on AC before admission	all patients had an acute thrombotic event, 76.2% AT and 23.8% DVT
Mao et al./2020 [55]	Retrospective observational case series	1/214	Not reported	5.7%	Not Reported	stroke was documented in 5.7%.
Bellosta et al./2020 [56]	Retrospective observational case series	1/20	Not reported	All patients included	25% on chronic AC due to AF	16.3% AT, significantly higher than pre-COVID
Perini et al./2020 [57]	Retrospective observational case series	1/4	Not reported	All patients included	LMWH prophylaxis	All patients despite thromboprophylaxis; 2 were young (53 and 37 years).
Oxley et al./2020 [58]	Retrospective observational case series	1/5	2 patients	All patients included	Not clearly reported	All patients had ischemic stroke and were under 50
Morassi et al./2020 [59]	Retrospective observational case series	1/6	83%	All patients included	3 patients (50%)	All patients developed stroke and 5 died

Legend: ICU-intensive care unit; AT-arterial thrombosis; AC-anticoagulant therapy; DVT-deep vein thrombosis; LMWH-low-molecular-weight heparin.

Several data sources additionally support the fact that most of the so-called PE occurred without an evident source of DVT, they should be better considered as primary, “in situ” pulmonary AT, a direct consequence of SARS-CoV-2 lung injury, determining thrombotic occlusions of smaller pulmonary arteries, which will cause the infarction of afferent pulmonary tissue [53,55,56]. This observation regarding the distinction between PE and primary in situ pulmonary AT could explain why PE is the main thrombotic event diagnosed in COVID-19 patients [47] and why ultrasound screening revealed a higher prevalence of VTEs than clinical assessment of asymptomatic patients [57]. Thus, in a noncritical care setting, PE in hospitalized COVID-19 patients is seemingly common, with nearly half of PE events being diagnosed after hospital admission [58]. Moreover, a recent meta-analysis of 11 cohort studies found that, despite anticoagulation, 23.9% of hospitalized COVID-19 patients developed VTE (11.6% had PE and 11.9% DVT), with ICU cases having a higher risk of developing VTE (30.4%), compared to the non-ICU group (13%) [45]. Furthermore, Klok et al., by analyzing 184 COVID-19 pneumonia ICU cases, all being under thromboprophylaxis, highlighted a 31% cumulative incidence of various vascular complications (PE, DVT, ischemic stroke, MI, or systemic arterial embolism). Computed pulmonary artery tomography and/or ultrasonography evidenced VTEs in 27% of cases and ATs in 3.7%, while the most frequently diagnosed thrombotic event was PE (81%). The main independent predictors for VTEs were age, with an adjusted hazard ratio (aHR) of 1.05/per year, and coagulopathy, certified by a spontaneous prolongation of the prothrombin time > 3 s or of the activated partial thromboplastin time (aPTT) > 5 s, with an aHR of 4.1 [47].

Conversely, a very extensive meta-analysis of 44 studies, on the topic of complications and mortality in 14,866 individuals hospitalized for a SARS-CoV-2 infection, determined a much lower incidence of 15% for VTE (only 3 studies reported VTE in a total of 318 patients); these data could have been influenced by cohort size and also by other elements such as heterogeneities between the selected studies increasing the risk of bias [9]. Of note, a very recent retrospective analysis of 2000 COVID-19 hospitalized cases revealed that only 36 (1.8%) of these cases developed VTE. Interestingly, almost all of the patients that developed VTE (35 out of 36), had been receiving LMWH. Despite the very low prevalence, overall mortality for COVID-19 complicated by VTE was 55.6% [40].

Referring strictly to DVT, another recent meta-analysis of 28 articles reported, using a random-effects model, a pooled estimated global prevalence of 16% (397 cases of confirmed DVT out of a total of 4138 COVID-19 patients evaluated), with variations regarding geographic region-a much higher pooled incidence of DVT was reported for the Chinese COVID-19 patient subgroup (30%), compared to Western countries (13%), and need for critical care (23% for patients requiring ICU admission as opposed to 5% for the non-ICU subgroup) [46].

This being said, as highlighted in the previous studies, VTE may still occur in noncritically ill COVID-19 individuals, so rigorous screening, risk assessment, and treatment protocols for VTE in COVID-19 would be essential, as these patients are much less likely to receive thromboprophylaxis. A retrospective cohort analysis of 289 noncritically ill hospital-admitted COVID-19 patients showed that 34.6% of patients needed diagnostic VTE imaging tests and these tests were positive for 17% of cases, while secondary endpoints showed a composite of in-hospital death or transfer to ICU occurring in 31% of evaluated cases, with a much higher prevalence rate in the VTE subgroup (47.9% vs. 27.9% non-VTE cases). Lack of thromboprophylaxis in the evaluated non-ICU COVID-19 cases was most likely a major determinant of VTE [50].

Regarding AT, a much lower prevalence in comparison to VTE has been reported among COVID-19 patients, and cases have consistently been reported since the beginning of the pandemic (3.7%, as mentioned above [36]), to date. An early Italian study, analyzing 388 consecutive, symptomatic, COVID-19 patients, reported low rates of occurrence for AT (stroke in 2.5% of cases, while acute coronary syndromes in 1.1% of patients) [49]. Another Italian study demonstrated a significantly increased incidence of acute limb ischemia (ALI) in the first 3 months of 2020, compared with those of 2019 (16.3% vs. 1.8%, respectively), most patients being elderly men with comorbidities [56].

As the pandemic unraveled, subsequent investigations reported alarming results regarding AT, as it became apparent that AT did not seem to occur only in elderly patients with predisposing comorbidities. Multiple, more recent case series reported strokes or ALI in younger, and generally healthy, people with SARS-CoV-2 infection [51,52,54,57,58,59]. Larger, retrospective studies also sustained that acute, cerebrovascular disease was not uncommon in patients suffering from COVID-19.

Nevertheless, more recently published data, from large health care systems, involving a larger population, showed that the actual prevalence of AT (thrombotic/embolic) is much lower than previously reported [43,44,60]. In their study, evaluating 1114 COVID-19 patients, Piazza et al. reported a prevalence of 0.1% and 1.3% for stroke and MI, respectively [43]. Comparatively, a more recent Chinese retrospective report, analyzing a consecutive series of 214, mild to severe, COVID-19 patients, found that 36.4% had neurologic manifestation (dizziness, headache, loss of sensory function), but stroke was only confirmed in 5.7% [55], a consistent and confounding higher rate than in Western reports. Several reasons were proposed to account for this dissonance, including differences in patient characteristics, diagnostic difficulties, and heterogeneity of independent adjudication of ATE diagnoses [43,60].

Even more worrisome are the reports of dramatic AT in young patients without significant comorbidities, with nonsevere disease phenotypes: common femoral artery thrombosis in a 24-year-old man and acute superior mesenteric artery thrombosis with subsequent small bowel necrosis in a 55-year-old man, both with mild COVID-19 [61,62].

### 3.2. Alterations of Hemostasis

An increased tendency to bleeding was reported somewhat later than thrombotic complications in patients with SARS-CoV-2 infection, especially in those hospitalized in ICU, contributing to the increased mortality of this illness [23,63]. Some researchers, Al-Shamkary et al. and Shah et al., reported an overall prevalence of 4.8–8% referring to hemorrhagic events, and of 3.5% for major bleedings [23,63], these complications being more frequent in withholder patients, mostly men, with comorbidities. As opposed to the numerous studies debating on the topic of thromboembolic events, we found fewer studies focusing on major bleedings and several case reports of hematomas (see Table 3).

### 3.3. Disseminated Intravascular Coagulopathy

Coagulopathy (DIC) as one of the most significant adverse prognostic signs, was described mostly among patients suffering from severe forms of the SARS-CoV-2 infection, hospitalized in intensive care units [18,25,64]. It was a common finding in most autopsy reports of patients who died from COVID-19 [65]. Coagulopathy results from the concomitant activation of coagulation and fibrinolytic pathways, probably due to severe sepsis and augmented levels of inflammatory mediators (“the cytokine storm”), leading to the consumption of coagulation factors and platelets as well [2,65].

### 3.4. Major Bleedings

Major bleeding, defined as fatal ones, occurring in critical organs (brain, eye, pericardium, retroperitoneal space, intra-articular, or intramuscular), or causing a decrease of the hemoglobin level of over 2 g/dL, or requiring at least two units of packed red blood cells transfusion, were reported in several studies debating over bleeding events observed in COVID-19 patients. Gastrointestinal bleeding, hemoptysis, oral mucosa bleeding, pulmonary, intracerebral, and renal hemorrhages, and bleeding from multiple cannulation sites have also been described [3,66,67].

A retrospective study from 2022 describes a much higher prevalence of bleeding events in ICU patients (26.7%) in comparison to 4.3% in non-ICU patients. Factors responsible for an increased thrombotic and hemorrhagic risk were advanced age, associated medical conditions, and some invasive procedures such as extracorporeal membrane oxygenation (ECMO) frequently used in ICU settings for severely ill patients [46].

A higher prevalence of gastrointestinal bleedings (GIB) was reported in two large multicenter studies, published in 2021, one in the USA realized on 11,158 patients who reported a prevalence of 3% for GIB which did not seem to be related to the use of ACT or/and antiplatelet therapy [68]. On contrary, another study, presenting data on 5344 subjects from the Lean European Open Survey on SARS-CoV-2 (LEOSS) and COKA registries, reported a total prevalence of GIB of 1.8%, higher in ICU patients (4.5%), but associated with the use of ACT [69].

Al Samkari et al., in a multicenter study, analyzed the prevalence of thrombotic and bleeding events in 400 hospitalized patients and observed an increased prevalence of bleedings of 7.6% in those hospitalized in ICU, with the risk being higher among male patients [23].

**Table 3:** Medical literature studies referring to hemorrhagic events – major bleedings.

Authors/Year	Study Type	Number of Studies/Patients	ICU	Prevalence of Hemorrhagic Events	Thromboprophylaxis	Comments
Valeriani et al./2022 [70]	Meta-analysis	9/5470	ICU/Ward	2.5% therapeutic 1.4% prophylaxis	100%	VTE 2.9% high dose and 5.7% prophylaxis
Wang et al./2022 [8]	Retrospective single-center study	138	ICU/Ward	4.3% Ward 26.7% in ICU	100%	16.67% VTE
Helmy et al./2022 [71]	Prospective observational study	114	ICU/Ward	15.8%	100%	13.2% VTE, 4.4% AT
Trindade et al./2021 [68]	Multicenter cohort study	11, 158	ICU/Ward	3%	Almost all	Gastrointestinal bleeding was associated with high mortality
Zellmer et al./2021 [69]	Retrospective multicenter cohort study	5344	ICU/non-ICU	1.8% 4.5% in ICU	Almost all	Higher mortality
Al Raizah et al./2021 [72]	Multicenter study	636	ICU/Ward	1.7% 9.4% in ICU	90%	1.8% VTE 2.2% AT
Halaby et al./2021 [73]	Retrospective cohort study	443	ICU	18.2%	100–76.2% therapeutic	Higher risk for therapeutic dose
Godier et al./2021 [74]	Retrospective study	56	All ICU	18%	100–75% therapeutic	29% VTE
Boira et al./2021 [17]	Series of cases	4	ICU/Ward	All	All	Equal prevalence between genders
Al-Samkari et al./2020 [23]	Retrospective multicenter study	400	ICU/Ward	7.6%/4.8%	All except one	6% VTE 2.8% AT
Fraisse et al./2020 [75]	Monocenter retrospective study	92	ICU	21%	All-prophylactic or therapeutic dose	40% VTE in ICU patients
Nadkarni et al./2020 [60]	Prospective multicenter study	153 of 4389	Non-ICU	5.6%	65.1% prophylactic or therapeutic dose	3% in subjects with therapeutic AC dose

Legend: ICU-intensive care unit; VTE-venous thromboembolism, AT-arterial thrombosis; AC-anticoagulant therapy.

Nadkarni et al. analyzed the characteristics of 153 patients with major bleedings in relation to the doses of anticoagulant drugs, concluding that in the case of treatment with therapeutic doses, the prevalence of bleeding events was 3.0%, in comparison to 1.959% in patients who received prophylactic anticoagulation and to 1.9% in those who did not receive such treatment at all [60]. Generally, bleeding rates were higher in individuals treated with LMWH in comparison with direct oral anticoagulants (2.6% and 1.3%, respectively). Gastrointestinal bleedings prevailed (50.7%), followed by mucocutaneous (19.4%), bronchopulmonary (14.9%), and then intracranial (6%) [60,68,69]. Because (sub)therapeutic doses of ACT have not decreased the risk of fatal or nonfatal thrombotic events while concomitantly elevating the hemorrhagic risk, their employment in noncritically ill COVID-19 patients should be carefully recommended [55].

### 3.5. Hematomas

Muscular and subcutaneous hematomas were seldom presented in the medical literature, mostly as case reports or series of cases, and were associated with increased morbidity and mortality [19,32,52,53,56]. Advanced age, male gender, and concomitant anticoagulant therapy were considered as risk factors for this complication.

The characteristics of intramuscular hematomas have been largely debated in an article by Abate et al., who analyzed ten patients with hematomas in addition to a literature review where he found an additional 40 cases, all being treated with various doses of LMWH [67]. They also concluded that hematomas occurred roughly 2–3 weeks after the onset of COVID-19 and even speculated that there could be a gender predisposition in the location of hematomas, the ileo-psoas disposition being more prevalent in males, compared to pectoral ones in females [52]. One of the earliest descriptions of multiple subcutaneous hematomas in an 84-year-old man suffering from a SARS-CoV-2 infection, was in 2020 in the paper of Matiolli et al. [76]. Probably, the higher number of cases was presented by Abate et al. (10 cases of 475 patients) who also advanced a prevalence of 2.1% of hemorrhagic events in patients treated with ACT, both in prophylactic, but mostly (60%) therapeutic doses. The authors concluded that the prevalence seems to be higher in males and is associated with a higher mortality rate [67].

There were also other relevant articles presenting similar cases of muscular or subcutaneous hematomas, occurring mostly in elderly men, usually during the acute infection and associated with ACT [19,21,77], but there are isolated remarks over such complications even during the recovery after cessation of ACT [78].

## 4. Discussion

The increased propensity for thromboembolic events accompanying the infection with the SARS-CoV-2 virus has been observed from an early stage of the COVID-19 pandemic. Precocious evidence supported the hypothesis of exacerbated thromboembolic processes in COVID-19, defined as immuno-thrombo-inflammation [79,80]. This process was considered to be a consequence of perturbed multiple biological pathways, including endothelial dysfunction, macrophage/monocyte, and neutrophiles activation, exacerbated immune responses of “the cytokine storm”, all of them impacting the coagulation cascade [24,32,66].

One of the first large meta-analyses investigating 14,866 patients from over 44 peer-reviewed studies and debating on the acute complications encountered in COVID-19 [9] advanced an overall prevalence of 15% for VTE and 6% for coagulopathy. Another early meta-analysis, analyzing data over thromboembolic events discussed in 42 studies, enrolling 8271 patients hospitalized for COVID-19, also stated an increased prevalence of 21% for DVT, higher in ICU cases (31%) with a pooled mortality rate of 23% in patients with VTE; although in autopsy cases, the rate of VTE was 35% [6]. The reported rate for ATs was an average of 2%, but 5% in ICU. Subsequently, numerous studies and several meta-analyses, by studying large populations from all over the world, advanced similar rates of around 30–35% for VTE occurring in ICU patients [10,34,36,46,47], but for non-ICU hospitalized individuals, the reported results varied largely (between 2.2 to 15%) [8,35,37,38,44], despite a thromboprophylaxis rate of 85–90%, depending on the evaluated subpopulation, and seemingly well-correlated with the severity of the disease and pre-existing metabolic and cardiovascular comorbidities [43]. A more recent meta-analysis of Overton et al., by studying large databases of hospitalized patients, but also of outpatients, reported diverse PE prevalence, varying from 0–1.1% in outpatients to 0.9–8.2% in hospitalized subjects and 1.8–18.9% in ICU patients [81].

More studies observed that thromboembolic complications occurred in the first week of the SARS-CoV-2 infection and were more frequent in patients admitted with severe respiratory failure being responsible for their increased mortality [10,36,46,47]. In some studies, a slightly higher prevalence of VTE and AT in elderly men has been documented [35].

Current guidelines do not recommend routine screening for VTE in all hospitalized patients. The employment of specific investigations, such as vascular compression ultrasound and echocardiography, should be recommended only in case of clinical suspicion [35].

It seems that over time the reported prevalence of thrombotic events shows a decreasing trend and may be less frequently encountered in COVID-19 caused by VOC/VOI than in the earlier strains, possibly also in connection with a larger use of the concomitant ACT during hospitalization, but also for prevention in high-risk outpatients, and during recovery as well, as recommended by several guidelines [29,30,33,35,37,38].

Shortly after initial reports on the increased incidence of thromboembolic complications in patients suffering from a SARS-CoV-2 infection, an elevated risk for bleeding complications has also been reported, mostly complicating the evolution of subjects hospitalized in ICUs and being responsible for their increased mortality [23,63]. The bleeding tendency in COVID-19 is quite rare, but it has been considered a manifestation of the so-called “COVID-19–associated coagulopathy” which encompasses immuno-thrombo-inflammation leading, subsequently, to coagulation disorders and imbalances in platelet production/disruption [32,66]. Often, an important contributing factor is the concomitant antithrombotic prophylaxis [23,32]. It has been observed that, generally, major bleedings occur somewhat later in the course of the disease, between the second and third week of evolution, as reported by Godier et al. in their research [74], while thromboembolic events are more frequent in the first week. Boira et al., in their study, confirmed this observation [17]. Most patients with bleedings were elderly, male gender prevailed, had comorbidities such as systemic hypertension and diabetes mellitus, and also had increased mortality [66].

Following these observations, an urgent need to prevent and treat these complications emerged. Starting from the premise that a two-way association exists between inflammation and the coagulation cascade, the so-called “immunothrombosis”, the administration of heparin appeared rational considering its property to block thrombin production, especially in the lung vasculature, thus limiting the progress of the SARS-CoV-2–driven inflammation [79,80]. However, in COVID-19, heparin offers additional advantages over other anticoagulants by multiple mechanisms such as anti-inflammatory proprieties through the inhibition of neutrophil chemotaxis and neutralization of several circulating cytokines (including IL-6), complement factor, and other acute-phase reactants. It is supposed that unfractionated heparin (UFH) might even exert some antiviral action, it improves microcirculation and could confer endothelial protection [29,30,33]. In COVID-19, the use of LMWH may offer certain advantages considering the less frequent administration and the lower incidence of heparin-induced thrombocytopenia. In contrast, UFH should be preferred in patients with renal impairment (creatinine clearance < 30 mL/min) and with an increased bleeding risk [30,33]. Some clinical studies suggest that, in COVID-19 patients, the plasma concentrations of direct oral anticoagulants (DOAC) could achieve variable levels due to the associated antiviral and, eventually, some experimental drugs. For example, the anticoagulation coronavirus (ACTION) randomized trial realized in 3331 patients from Brasil, aimed to assess whether in-hospital anticoagulation with DOAC in stable patients or enoxaparin in therapeutic dose in ICU subjects, followed by DOAC for 30 days, in comparison to in-hospital prophylactic anticoagulation with UFH, decreases the duration of hospitalization, the need for additional oxygen support or mortality, as well as the prevalence of clinically relevant nonmajor bleeding, but the results did not show the superiority of DOAC, or enoxaparin followed by DOAC versus prophylaxis with UFH [82].

Until further recommendations, in the absence of contraindications, actual guidelines of the American Society of Hematology favor the employment of ACT in therapeutic over prophylactic doses in patients with COVID-19 without suspected or confirmed VTE. The panel insists on the necessity of an individualized evaluation of thrombotic and bleeding risk. The panel highlighted that heparin, either UFH or LMWH, could be superior to other anticoagulants [30]. A recent review [83] summarized the recommendations of the American Society of Hematology in comparison to those of the International Society for Thrombosis and Hemostasis and of the American College of CHEST Physicians for prophylactic and therapeutic anticoagulation, especially since the last two propose an extended anticoagulation of up to 14–30 days during recovery, with LMWH or DOAC, in patients that meet high-risk criteria, especially those with severe forms of COVID-19, treated in ICUs [84] or those with malignancies where special attention should be granted to the increased risk of thromboembolic events [85]. Patients with absolute contraindications to pharmacological thromboprophylaxis may be the candidates for mechanical thromboprophylaxis with intermittent pneumatic compression.

Even more concern was raised by more recent observations over a higher prevalence of thromboembolic events after vaccination. This controversial topic is debated in several articles. A recent meta-analysis of 20 studies [86] evidenced 286 thromboembolic events in a younger population (48.5 ± 15.4 years), mostly women (67.4%), predominantly after 10.8 ± 7.2 days since vaccination with the Astra Zeneca vaccine (93.7%). VTE was more frequent (74.8%) than AT (27.9%), and concomitant thrombosis was observed in 15.4% patients. Most patients also had associated coagulation disturbances, thrombocytopenia (49%), and antiplatelet factor 4 antibodies (78.6%) prevailing.

Study limitations: considering the immense amount of medical literature written on the topic of COVID-19, particularly on the increased risk of thromboembolic events, although in our study we researched several databases on scientific articles employing specific keywords, we elaborated a literature review by selecting only papers debating over hospitalized patients considered by us as presenting representative results concerning the prevalence, outcome, and therapy of thromboembolic complications frequently associated with bleeding events.

## 5. Conclusions

Thromboembolic events are frequent complications encountered in patients infected with the SARS-CoV-2 virus, especially in those with severe forms and comorbidities. Both thromboembolic events and hemorrhagic complications aggravate the evolution of these patients, representing significant negative prognostic factors and increasing the morbidity and mortality associated with COVID-19. For their prophylaxis/treatment, anticoagulant therapy is recommended, thus increasing the risk of bleeding. This is the reason why it should be individualized for each patient.

## Data Availability

Not applicable.
